# Mycophenolate-induced colitis in a patient with lupus nephritis: a case report and review of the literature

**DOI:** 10.1186/s13256-024-04539-7

**Published:** 2024-05-01

**Authors:** Ziyad Alakkas, Abdulaziz M. Gari, Sara Makhdoum, Eman A. AlSindi

**Affiliations:** 1 Rheumatology Unit, Internal Medicine Department, King Abdul-Aziz Specialist Hospital, Taif, Saudi Arabia; 2 Rheumatology Unit, Internal Medicine Department, King Fahad Hospital, Jeddah, Saudi Arabia; 3 Histopathology Department, King Fahad Hospital, Jeddah, Saudi Arabia

**Keywords:** Mycophenolate mofetil, Colitis, MMF, Case report

## Abstract

**Background:**

Mycophenolate mofetil (MMF) is an immunosuppressive drug that is frequently prescribed to patients with rheumatological diseases. MMF’s side effects include abdominal discomfort, nausea, vomiting, and other gastro-intestinal side effects, which typically appear in the first few months of treatment. However, late-onset diarrhea does not rule out the presence of MMF-induced colitis, which can be misdiagnosed since it is linked to a broad range of histopathological characteristics, including alterations that resemble inflammatory bowel disease, graft-versus-host disease, and ischemia. The differences in treatment response may be explained by the complexity of the histopathologic characteristics.

**Case presentation:**

Here we present a case of a 29-year-old Arabian female with lupus nephritis who started on MMF as induction therapy. In two months, the patient was presented to the Emergency Department with diarrhea and manifestations of severe dehydration. Infectious diseases and adverse drug events were suspected, so the patient was admitted for further workup, and MMF was stopped. The patient was diagnosed with MMF-induced colitis based on colonoscopy and histological findings. Fourteen days after stopping MMF, she was within her baseline.

**Conclusion:**

The purpose of this paper is to report a case of early-onset MMF-induced colitis in a patient with lupus nephritis who had started MMF as induction therapy. A review of the available literature on this uncommon immunosuppressive effect is also presented.

## Introduction

Mycophenolate mofetil (MMF) is widely used as an immunosuppressive agent for various inflammatory and/rheumatic conditions, including lupus nephritis and organ transplantation. Mycophenolic acid is an active metabolite of MMF that reversibly inhibits inosine monophosphate (IMP) dehydrogenase, preventing purine synthesis in T and B cells [1, 2]. Dose modification or even discontinuation of MMF is quite common due to adverse effects, especially gastro-intestinal side effects, which occur in nearly 45% of cases [2]. Enterocytes are particularly susceptible to the antimetabolic effects of MMF due to their reliance on the de novo process of purine synthesis. This prevents the growth and reproduction of small bowel epithelial cells, which disrupts fluid absorption and causes diarrhea [3, 4]. However, MMF is a well-tolerated therapy in general.

One of the major adverse effects of MMF is colitis, which can lead to serious complications that include perforation, bleeding, and hospitalization. In recent years, several studies have investigated the factors associated with MMF-induced colitis. One study evaluated the incidence of gastro-intestinal complications following kidney transplant and showed that MMF-induced colitis was the most common type of colitis, occurring in 6–9% of patients, and the most common symptom was diarrhea [5, 6]. MMF is one of the common immunosuppression medications for rheumatological disease and has been used for the last two decades with very good outcomes in terms of different aspects and system involvement.

There are only a few reports of patients who developed MMF-related colitis (Table 1). A small retrospective study evaluated 11 patients with rheumatologic disease who had been treated with MMF and found that only one patient had medication-related colitis [7]. We report a case of a young female who had systemic lupus erythematosus and lupus nephritis and presented with abdominal pain and diarrhea. We also discuss the challenges in the diagnosis of MMF-induced colitis.

**Table 1:** Lists of the publication papers of mycophenolate mofetil induced colitis for the last 15 years

	Year	Type of study	Gender	MMF dose	Duration of MMF	Disease background	Histologic finding	Timing of symptoms resolution	References
1	2023	Case Report	Female	1g BID	3 months	Kidney transplant	Increased crypt apoptosis, mild architectural disarray, and focal crypt attenuation	N/A	[15]
2	2021	Cohort 4 out of 10 patients	3 Females 1 Male	3 on MMF [1–3 g/day] 1 on MFS	2 weeks–8 months	APECED	All IBD-Like Pattern 3 overlapped with GVHD 1 Absent enterocyte apoptosis	1–2 weeks	[16]
3	2021	Case report	Female	N/A	N/A	Kidney Transplant	Dilated crypts with edema and numerous eosinophils in lamina propria. Rare Pigmented Macrophages	1 week	[17]
4	2020	Case report	Male	1 gm BID	8 months	Lung Transplant	Mild crypt architectural distortion with crypt cell apoptosis	N/A	[18]
5	2018	Case report	Male	500 mg BID	6 months	Heart Transplant	Focal crypt abscesses with occasional apoptosis of epithelial cells, frequent tangible body macrophages and eosinophils within the lamina propria	5 weeks	[19]
6	2018	Case report	Female	N/P	2 months	Systemic Sclerosis	Ulceration, granulation tissue, and hyalinized mucosa and submucosa	5 days	[20]
7	2017	Case report	Male	1 g/day	2 years	Kidney Transplant	Mild crypt distortion	N/A	[21]
8	2016	Systemic review 544 patients	30% Female	N/A	990 days (range 3- 5760)	N/A	N/A	20 days (range 1–45)	[22]
9	2016	Case report	Female	N/A	10 years	Lupus Nephritis	Crypt atrophy and increased crypt apoptosis	3 days	[23]
10	2015	Retrospective	21 Male (58%)	N/A	N/A	Kidney Transplant (58%)	Acute colitis like (50%) IBD- like (36%) GVHD-like (8.3%) Ischemia-like (5.6%)	N/A	[6]
11	2014	Case report	Male	1 g BID, escalated to 1.5 g BID 4 months pre presentation	28 months	Autoimmune autonomic dysfunction	Dilated damaged crypts, eosinophilic epithelial changes, and crypt abscesses with apoptotic bodies	5 weeks	[24]
12	2012	Case report	Male	N/A	50 months	Kidney transplant	Mild crypt architectural distortion Increased number of inflammatory cells many neutrophils	5 days	[25]
13	2012	Case report	Male	N/A	8 months	liver transplant	Crypt cell apoptosis with focal crypt distortion and dropout	1 week	[26]
14	2012	Case report	Female	1 g/day	1 year	Mixed connective tissue disease	Apoptosis, crypt distortion and abscess	5 days	[27]
15	2010	Case report	Female	1 g/day	6 years	Kidney Transplant	Ulceration with mixed cellular inflammatory infiltrate	3 days after infliximab infusion	[28]
16	2009	Retrospective of 11 patients with rheumatologic disease on MMF	One patient with colitis Female	1–3 g	22 months	Polymyositis	N/A	N/A	[7]

*APECED* autoimmune polyendocrinopathy-candidiasis-ectodermal dystrophy, *MMF* mycophenolate mofetil, *MPS* Mycophenolate Sodium, *IBD* inflammatory bowel disease, *GvHD* Graft versus host disease, *N/A* data is not available

Written consent was obtained from the patient, and ethical approval was provided by the Institutional Review Board (IRB) of the Study and Research Department of King Fahad Hospital, Jeddah.

## Case presentation

The patient was a 29-year-old Arabian woman with a known case of systemic lupus erythematosus, which had been diagnosed 5 years prior based on the criteria of the European League Against Rheumatism (EULAR)/American College of Rheumatology (ACR). She was started on hydroxychloroquine at 200 mg orally once per day, and she had no comorbidities except for hypothyroidism.

The patient had not been followed up due to the COVID-19 pandemic, but in January 2023, she presented to the clinic with an incidental lab result showing a creatinine level of 4.3 mg/dL. Thus, a renal biopsy was planned, and she was diagnosed with class IV lupus nephritis. She received pulse methylprednisolone therapy at 500 mg intravenously for 3 days, which was then switched to a tapering dose of prednisolone. Induction therapy using MMF was initiated at 500 mg orally twice daily then titrated up weekly until she was discharged to home on 1.5 g orally twice per day, which is the maximum recommended dose of induction phase for lupus nephritis.

After 2 months, the patient presented to the emergency department with complaints of nausea, vomiting, and left-sided abdominal pain associated with diarrhea 5–6 times per day, which was watery but contained no blood or mucus. Her symptoms started at just 2 weeks after starting MMF therapy and had progressed over the last month. She denied having fever, weight loss, or night sweats. Upon physical examination, she was alert and oriented but in pain. The abdomen was tender, but there were no signs of peritonitis. The rest of the physical examination was unremarkable apart from Cushingoid face. She was afebrile, and her blood pressure, heart rate, respiratory rate, and oxygen saturation were within normal ranges. Her weight was 90 kg.

Upon admission, MMF was promptly discontinued due to the possibility that it might have led to an infection. The immediate care involved the delivery of intravenous fluids, a low-residue diet, analgesic medications like intravenous acetaminophen, and antispastic treatments. Routine blood tests performed at admission indicated leukopenia 3.800 × 10^6^/L normal range (4–11 × 10^6^/L), elevated creatinine 6.3 mg/dL normal range (07–1.3 mg/dL) while her baseline of creatinine was 2 mg/dL and GFR 60 ml/min, and noticeably increased inflammatory markers (C-reactive protein 32 mg/dL normal range < 5 mg/dL, erythrocyte sedimentation rate 40 mm/hour normal range < 20 mm/hour). The C3 and C4 complement levels were 0.6 g/L (0.8–1.6 g/L) and C4 0.18 g/L (0.20–0.65 g/L), respectively. Stool analysis indicated + 2 pus cells and a negative culture. Urine analysis indicated a protein level of + 1 with no red blood cell crystals or casts. An abdominal ultrasound was performed, but the result was unremarkable.

**Fig 1:**
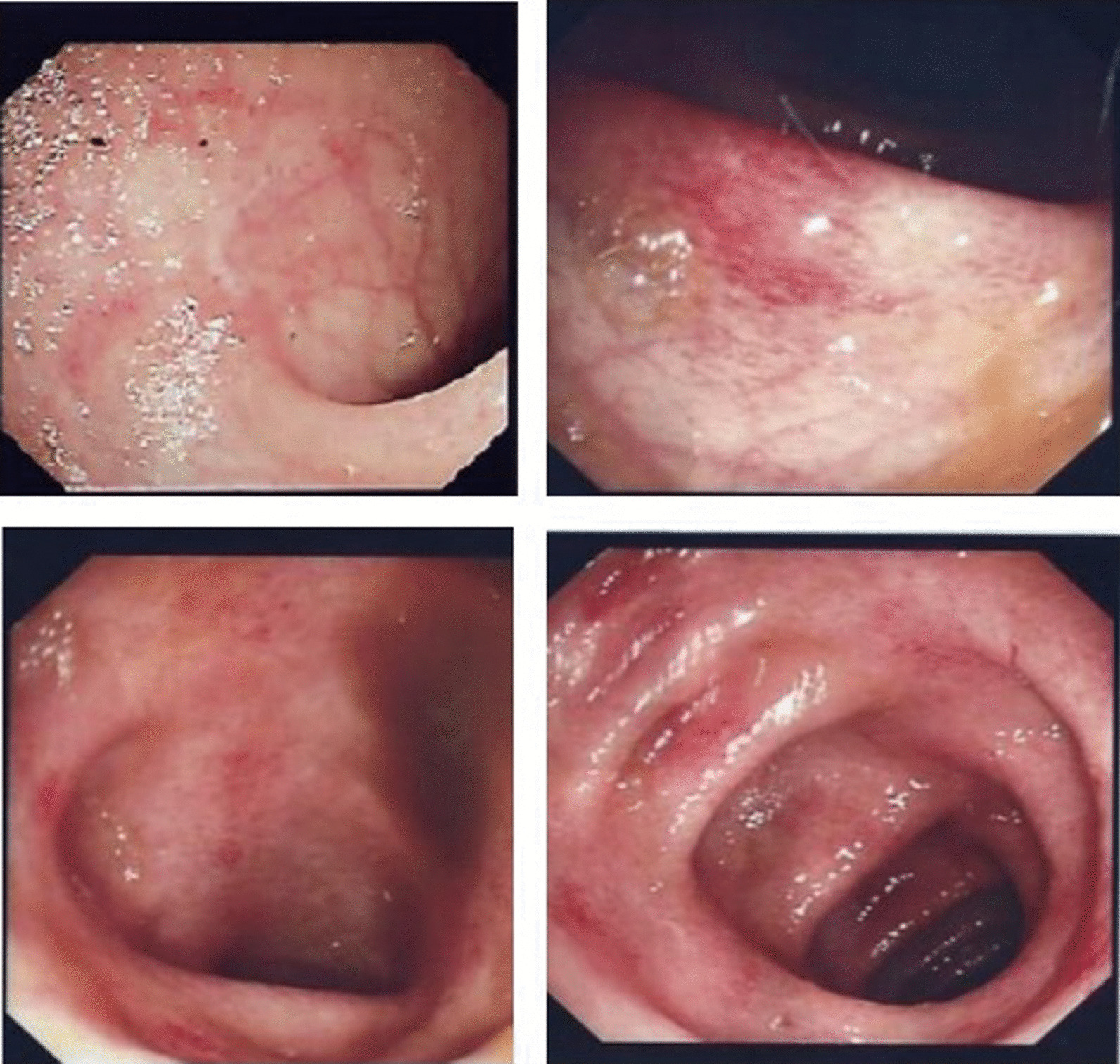
Colonoscopy shows hyperemic mucosa with some superficial ulcers from sigmoid up to the cecum, while the terminal ileum shows superficial ulceration with some area of inflammatory patches

Later, computed tomography scan was performed, which did not reveal any other abnormalities and confirmed the ultrasound results. A colonoscopy showed erythematous patches with few erosions and rectal-sparing colitis. Multiple biopsies been taken (Fig. 1). Infectious colitis, drug-induced colitis, newly diagnosed inflammatory bowel disease (IBD), gastro-intestinal involvement associated with systemic lupus erythematosus, and mesenteric ischemia were all considered in the differential diagnosis. Cytomegalovirus (CMV) infection is the main concern among infectious causes of colitis in patients with impaired immune systems, and its possible endoscopic findings include diffuse erythema, ischemia, erosions, and ulcers.

**Fig. 2:**
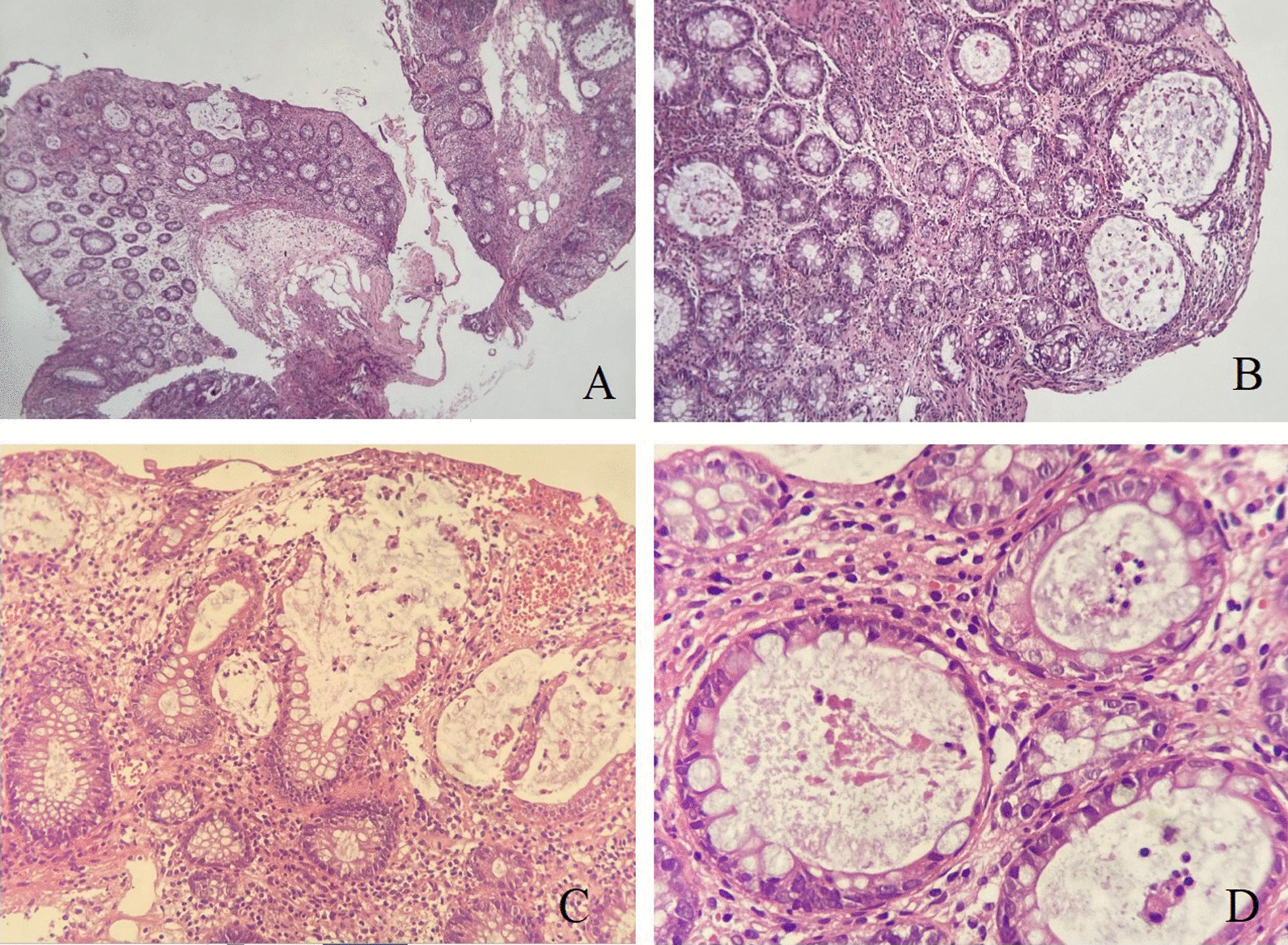
Mycophenolate Mofetil induced colitis. **A** Colonic biopsy with mild architectural distortion, crypt hyperplasia and lamina propria inflammation (HEx 4 ×). **B** Higher magnification (HE 20 ×) show dilated colonic glands. **C** Destructed ruptured colonic glands with mucin spillage (HE × 20 ×). **D **Acute inflammation within the glands (cryptitis), at high power magnification (HE x 40 ×)

The endoscopic appearance of drug-induced colitis can resemble that of ulcerative colitis, infectious colitis, and ischemic colitis. Non-steroidal anti-inflammatory drugs (NSAIDs) are the most common cause of drug-induced colitis, but MMF was probably involved in the present case. Rectal sparing almost always occurs in MMF-induced colitis. Microscopically, the colonic mucosa displayed a mild architectural distortion, with ruptures of few dilated glands. Mild cryptitis is observed. However, no obvious apoptosis seen as been previously described in few cases of MMF induced colitis. There were no evidence of viral cytopathic changes, granuloma, dysplasia or malignancy (Fig. 2A–D),

Following the cessation of MMF, the gastro-intestinal symptoms and the biomarkers for systemic inflammation gradually subsided and returned to base line levels after 14 days without further therapy, thus supporting the suspicion of drug-induced colitis. The patient was discharged 24 days after admission, MMF was discontinued, and the Euro-Lupus protocol was started with cyclophosphamide as induction therapy for lupus nephritis. The patient has been followed up closely and has shown improvement of active and chronic issues with 6 doses of cyclophosphamide completed. Azathioprine with a low dose of steroid was initiated. There have been no other gastro-intestinal manifestations.

## Discussion

MMF is an immunosuppressive medication that was first used to decrease the risk of organ rejection after transplantation, but now, it is also being used to treat patients with autoimmune systemic disorders, including systemic lupus erythematosus. The target dose of MMF for the treatment of Lupus Nephritis is 2–3 g per day in combination with glucocorticoids especially for those high-risk patients for kidney failure including reduced GFR. Dosage may need to be adjusted according to adverse events, toxicity, efficacy and MPA blood level [according to 2019 European Leage Against Rheumatism and European Dialysis and Transplant Association (EULAR/ERA-EDTA) recommendation for the management of Lupus Nephritis]. General recommendation to not exceed 2 g in patients with chronic renal failure with GFR less than 25 mL/min.

As far as we are aware, there have been only a few documented cases of colitis caused by MMF in a patient with a rheumatological condition. The onset of MMF-induced colitis in a patient with lupus nephritis, sclerosis, mixed connective tissue disease (MCTD), and polymyositis has previously been documented by other authors (Table 1). In transplant recipients, however, MMF is a well-known trigger of drug-induced colitis [6].

Mycophenolate targets tissues with fast cell division and reliance on purine synthesis. Lymphocytes and gut cells are the two main organs in which regeneration is dependent on this system. Immunosuppression results from lymphocytes (B and T cells) being more dependent on this route (by 90%) [8]. The blood level of mycophenolic acid is directly inversely correlated with mycophenolate’s adverse effects [9]. Since 50% of enterocytes rely on the mycophenolate-targeting mechanisms, it is believed to explain why 45% of patients experience gastro-intestinal side effects, including simple diarrhea, esophagitis, gastroesophageal reflux disease, enteritis, and colitis, as in our patient [2]. The most typical gastro-intestinal mucosal pattern associated with MMF is mucosa that seems normal [10]. The histological changes in patients receiving MMF have mostly been classified in many studies as normal or near normal in around one-third of cases, followed by changes resembling IBD, graft-versus-host disease (GVHD), self-limited colitis, and ischemia [11–13]. Another study reported histological results that were in line with an acute colitis-like pattern in half of cases as being the most common, followed by IBD-like pathologic findings in 36% of cases, ischemia-like characteristics in 5.6% of cases, and GVHD-like abnormalities in 8.3% of cases [6]. Examples of specific histological characteristics of MMF-related colitis include crypt architectural disarray, increased lamina propria inflammation, dilated damaged crypts, increased crypt epithelial apoptosis, and GVHD-like alterations [14].

The wide morphological spectrum documented in MMF-induced colitis includes features that can lead to misdiagnosis and delayed intervention. Therefore, it is essential to discuss the clinical history of MMF therapy with pathologists and to take this diagnosis into consideration, regardless of the length of therapy, given the variations in the therapeutic management and prognosis of these disorders. The most frequent indication for a colonoscopy referral for patients on MMF medication is diarrhea. Nearly half of such patients have normal colonoscopy results. Other endoscopic findings include erythema (33%) and erosions/ulcers (19%), which indicate a need for routine biopsies to help with confirmation of the diagnosis [6].

Treatment options range from stopping MMF use to using specialized immunosuppressive medications to correct the histological pattern replicated by MMF-induced colitis. There are no recommendations available to help clinicians treat colitis induced by MMF. Case reports have frequently shown that after stopping MMF, diarrhea symptoms improve within a week. In another study, after unsuccessful attempts with MMF cessation, a patient was given 50 mg of intravenous steroids daily for two weeks and a single infusion of 5 mg/kg of infliximab, which led to decreased stool frequency within three days after infusion [28].

## Conclusion

It is well known that MMF causes drug induced colitis with a variety of patterns and clinical manifestations. When caring for people with autoimmune systemic disorders, colitis should be recognized as a rare side effect of MMF therapy. It is necessary for physicians to be aware that discontinuing the medicine is typically effective without the need for extra treatments.

## Data Availability

Not applicable.

## Abbreviations

MMF: Mycophenolate mofetil
MPS: Mycophenolate sodium
IMP: Inosine monophosphate
GI: Gastro-intestinal
IBD: Inflammatory bowel disease
CMV: Cytomegalovirus
APECED: Polyendocrinopathy-candidiasis-ectodermal dystrophy
